# Marked Response to Larotrectinib in Pelvic Spindle Cell Sarcoma With Concurrent TPM3-NTRK1 Fusion and CDK4/MDM2 Amplification: A Case Report

**DOI:** 10.1200/PO.22.00528

**Published:** 2023-01-18

**Authors:** Wen-Kuan Huang, Shih-Chiang Huang, Chien-Ming Chen, Po-Hung Lin, Chiao-En Wu, Cheng-Keng Chuang, Chun-Nan Yeh

**Affiliations:** ^1^ Division of Hematology/Oncology, Department of Internal Medicine, Chang Gung Memorial Hospital at Linkou, Chang Gung University, College of Medicine, Taoyuan, Taiwan; ^2^ Department of Anatomic Pathology, Chang Gung Memorial Hospital at Linkou, Chang Gung University, College of Medicine, Taoyuan, Taiwan; ^3^ Department of Medical Imaging and Intervention, Chang Gung Memorial Hospital at Taipei, Chang Gung University, College of Medicine, Taoyuan, Taiwan; ^4^ Division of Urology, Department of Surgery, Chang Gung Memorial Hospital at Linkou, Chang Gung University, College of Medicine, Taoyuan, Taiwan; ^5^ Department of Surgery and GIST Team, Chang Gung Memorial Hospital at Linkou, Chang Gung University, College of Medicine, Taoyuan, Taiwan

## Background

Primary pelvic sarcoma is rare and often involves adjacent organs including rectum and urinary bladder because of the anatomic constraints of the pelvis.^1^ This may impede the attempts to achieve wide excision with tumor-free margins. Here, we reported that perioperative targeted therapy against tyrosine receptor kinase is a promising therapeutic approach to significantly improve surgical outcomes in huge pelvic spindle cell sarcoma with neurotrophic tyrosine receptor kinase (*NTRK*) gene fusion.

## Case Presentation

**FIG A1.**
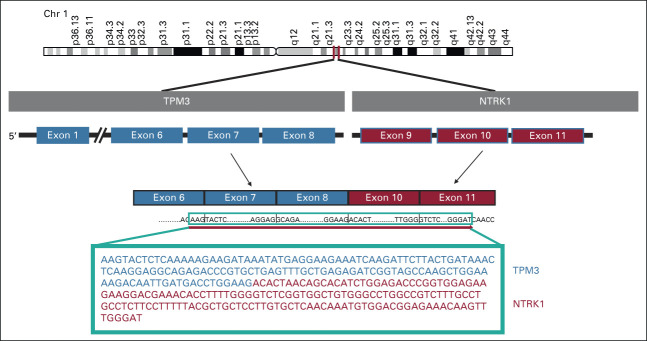
Scheme of *TPM3::NTRK1* rearrangement in this case. The position of the breakpoints leading to the *TPM3::NTRK1* rearrangement is with reference to genome reference hg38 version.

A 43-year-old man presented with 2-week history of urinary retention and difficulty in defecation. Initial computed tomography revealed a huge solid pelvic mass (length [L] × width [W] × height [H]: 10.0 × 9.0 × 9.8 cm) displacing the rectum posteriorly and the urinary bladder and prostate gland superiolaterally. Percutaneous transrectal biopsy revealed a spindle cell tumor, which was focally positive for CD117 and DOG-1 and negative for smooth muscle actin, desmin, and S100 on immunohistochemical stains. Under the impression of rectal GI stromal tumor, the patient started imatinib (400 mg once daily). After 2 months of treatment, follow-up magnetic resonance imaging (MRI) showed marked progression of the tumor (L × W × H: 13.0 × 10.6 × 14.0 cm). Given the state of imatinib failure and unusual focal CD117/DOG1 expression for GI stromal tumor,^2^ we screened this patient with pan-TRK immunohistochemical staining, which was diffusely positive. The tumor specimen was sent for further analysis for next-generation sequencing (NGS; ACTOnco+, ACT Genomics), which mainly disclosed *TPM3::NTRK* fusion (Appendix Fig A1), and *MDM2/CDK4* gene amplification. Neither cKIT nor PDGFRA had mutations. In addition, both *MDM2* and *CDK4* stains were diffusely positive. Altogether, *NTRK* fusion–positive spindle cell neoplasm or dedifferentiated liposarcoma was suspected.

Written informed consent from this patient was obtained, and this study was approved by the institutional review board of the Chang Gung Memorial Hospital. We obtained institutional written consent from the patient for publication of his data under anonymized format.

**FIG 1.**
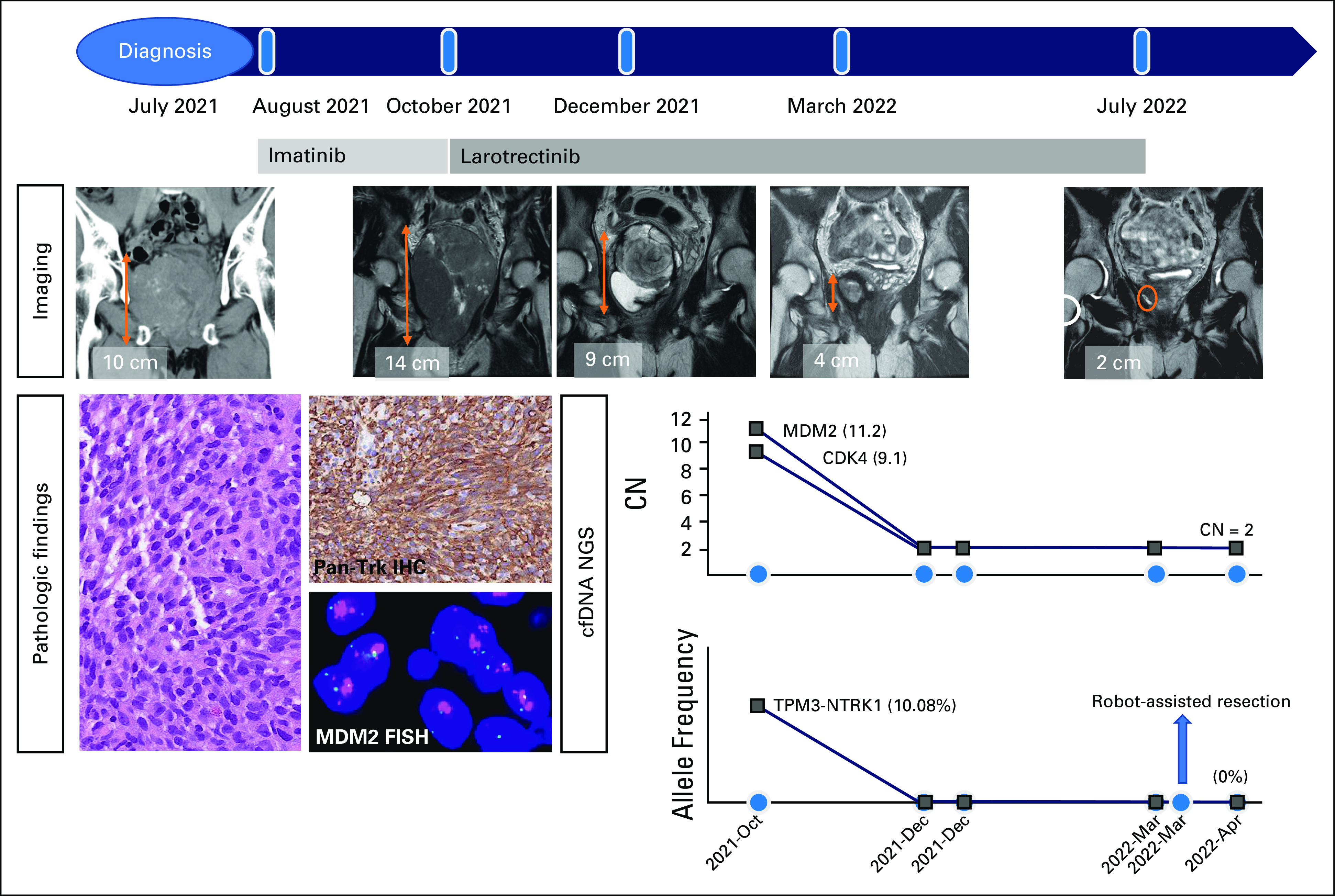
Course of the treatment history with serial images, pathologic findings (IHC and FISH), and cfDNA NGS in this patient with a pelvic sarcoma harboring *NTRK* fusion and *MDM2/CDK4* amplification. cfDNA, cell-free DNA; CN, copy number; FISH, fluorescence in situ hybridization; IHC, immunohistochemistry; NGS, next-generation sequencing.

He commenced on larotrectinib (100 mg, orally, twice daily) since November 2021. To assess disease dynamics in a longitudinal manner, we adopted a cell-free DNA (cfDNA) NGS assay (AlphaLiquid-100, IMBdx) for serial monitoring of 106-gene genetic alterations including *NTRK* fusion, *MDM2*, and *CDK4* amplification. The analysis of the cfDNA NGS panel before treatment with larotrectinib revealed both *TPM3::NTRK1* fusion and *MDM2/CDK4* amplification (Fig 1), reliably reflecting the genetic alterations of the original pelvic tumor tissue.

Treatment with larotrectinib was well tolerated and led to rapid clinical improvement within two weeks. After 8 weeks of larotrectinib, follow-up MRI revealed tumor necrosis and regression in size (L × W × H: 8.4 × 6.4 × 9.4 cm; Fig 1). After 18 weeks, further tumor regression (L × W × H: 4.9 × 3.8 × 4.4 cm) achieved best overall response of partial response according to RECIST 1.1 criteria. The tumor was closely adherent to the prostate gland and right seminal vesicle but separate from the rectum and urinary bladder. The successive cfDNA assays at 4 weeks, 8 weeks, and 18 weeks showed that *NTRK* fusion and *MDM2/CDK4* amplification were undetectable. He underwent robot-assisted radical resection where the prostate and prostatic urethra were partially resected. Macroscopic examination of the specimen revealed a 5.5 × 3.8 × 3.6 cm yellowish mass inseparable from the prostate. Microscopic examination showed extensive inflammation and fibrosis with only < 10% of residual viable tumor, representing good treatment response. No area of well-differentiated liposarcoma was identified peripherally. The encircled area of viable tumor cells (< 5% of the whole slide) was macrodissected for NGS analysis (ACTOnco+, ACT genomics). We did not detect *NTRK* fusion or *MDM2/CDK4* amplification in the residual tumor tissue, which was in line with the results of cfDNA-based assay at day 14 after resection. Larotrectinib was continued after operation, and he remained disease-free for a follow-up of 4 months (Fig 1).

## Discussion

Interestingly, we reported here this case who had a spindle cell sarcoma with both *MDM2/CDK4* amplification and *NTRK* fusion, which was also found in a recent report with three cases.^3^ Whether patients with tumor harboring *MDM2/CDK4* amplification need screening for *NTRK* fusions remains unclear and warrants further investigation. Of note, those three cases, including two dedifferentiated liposarcoma and one intimal sarcoma, did not receive *NTRK* inhibitor.^3^ Here, we reported one patient with sarcoma that harbors both *NTRK* fusion and *MDM2/CDK4* amplification, which responded well to the *NTRK* inhibitor.

The efficacy of *CDK4* inhibitors such as palbociclib and abemaciclib in advanced liposarcoma with *MDM2/CDK4* amplification is modest in early trials and real-world settings,^4-6^ suggesting a cytostatic rather than cytotoxic effect. *NTRK* inhibitors exhibited more promising and durable responses in sarcoma harboring *NTRK* fusion.^2^ Therefore, larotrectinib was selected to maximize the tumor response for following surgical resection. The marked response in this case suggests that *NTRK* fusion is the oncogenic driver of this pelvic sarcoma regardless of the presence of *MDM2/CDK4* amplification.

Our case demonstrated the potential utility of cfDNA NGS for serial and real-time monitoring of tumor response during the treatment period of larotrectinib. Of note, we were curious whether *MDM2/CDK4* amplification would be persistently detected by cfDNA NGS despite inhibition of *NTRK* fusion, which may indicate the pre-existing resistant clones. Surprisingly, both *NTRK* fusion and *MDM2/CDK4* amplification have been undetected after 5-week treatment of *NTRK* inhibitor, which was confirmed by the NGS testing of the resected tumor sample. These findings support the advantage of liquid biopsy recapitulating tumor heterogeneity.

In summary, a patient with locally advanced pelvic sarcoma responded well to neoadjuvant larotrectinib and successfully received subsequent surgical resection for curative intent. He remains disease-free for 8 months, to date, after 4-month adjuvant larotrectinib. To the best of our knowledge, this is the first case of a spindle cell sarcoma with both *TPM3::NTRK1* fusion and *MDM2/CDK4* amplification showing a significant response to *NTRK* inhibitor, suggesting *NTRK* fusion plays an oncogenic role in this sarcoma. Of note, this patient did not need to receive pelvic exenteration with urinary or fecal diversion because of marked response to *NTRK* inhibitor, which demonstrated that a precision oncology approach with a multidisciplinary team is important for the diagnosis and treatment of these rare sarcomas.

## Data Availability

Immunohistochemistry staining and raw sequencing data are available from the corresponding author upon reasonable request.
